# Volmer–Weber growth of nano-island heterostructures on spinel cathodes: a route to stable high-voltage lithium-ion batteries

**DOI:** 10.1039/d5sc07152f

**Published:** 2025-10-31

**Authors:** Gui Chu, Yuanqin She, Aoyu Huang, Qingquan Ye, Yimei Deng, Tongen Lin, Yongqi Sun, Tobias U. Schülli, Lianzhou Wang, Xiaobo Zhu

**Affiliations:** a College of Materials Science and Engineering, Changsha University of Science and Technology Changsha 410114 P.R. China xbzhu@csust.edu.cn; b Nanomaterials Centre, School of Chemical Engineering, and Australian Institute of Bioengineering and Nanotechnology, The University of Queensland Brisbane QLD 4072 Australia tongen.lin@uq.edu.au l.wang@uq.edu.au; c Dept. of Applied Biology and Chemical Technology, The Hong Kong Polytechnic University Hung Hom Kowloon Hong Kong SAR China; d School of Metallurgy and Environment, National Center for International Cooperation of Clean Metallurgy, Central South University Changsha 410083 P.R. China; e ESRF—The European Synchrotron 38000 Grenoble France schulli@esrf.fr

## Abstract

Engineering stable electrode–electrolyte interfaces is paramount for the operation of high-voltage lithium-ion batteries. Here, we demonstrate the spontaneous formation of a uniform zirconia nano-island architecture on 5V-class spinel crystallites by introducing a trace zirconium precursor. This phenomenon, rationalized as a Volmer–Weber growth mechanism, is thermodynamically driven by the immiscibility of Zr and a large lattice mismatch (∼10%) between the surface-templated cubic ZrO_2_ and the spinel substrate, which prevents further coalescing into large aggregates or a continuous film. Crucially, this discrete nano-island architecture offers a unique solution to a long-standing coating dilemma, overcoming the transport-blocking nature of pinhole-free films whilst offering comprehensive surface protection. It simultaneously enhances surface conductivity and stability as well as anchors a robust cathode–electrolyte interphase. As a result of this multifunctional interface, the optimized cathode exhibits outstanding electrochemical stability, retaining 90.8% of its capacity after 1000 cycles in half-cells and 77.5% after 500 cycles in pouch cells paired with graphite anodes.

## Abstract 2

A uniform zirconia nano-island architecture is grown on a high-voltage spinel cathode *via* a thermodynamically driven Volmer–Weber growth. It provides exceptional stability by resolving the dilemma of conventional surface coatings.

## Graphical abstract

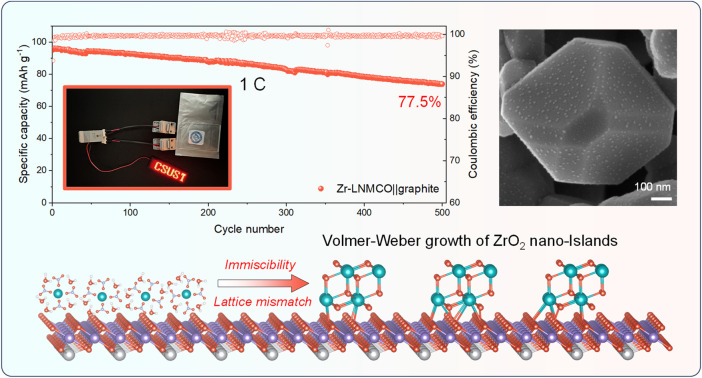

## 1 Introduction

Lithium-ion batteries (LIBs) dominate the battery market due to their high energy density and excellent rechargeability.^1,2^ The increasing adoption of LIB-powered electric vehicles as a primary strategy for mitigating greenhouse gas emissions has escalated demand, necessitating advancements in electrochemical performance as well as the utilization of sustainable materials.^3–6^ 5V-class spinel LiNi_0.5_Mn_1.5_O_4_ (LNMO), offering considerable energy density with a cobalt-free, nickel-lean composition, is positioned as a promising cathode for next-generation LIBs.^7–13^ However, the practical application of LNMO is impeded by accelerated electrochemical degradation and cell failure. The high operating voltage exceeds the stability window of standard carbonate-based electrolytes, triggering electrolyte oxidation and the generation of corrosive acidic species.^14^ These species attack the cathode–electrolyte interphase (CEI) and the cathode surface, causing transition metal (TM) dissolution, which accelerates capacity fade, particularly under elevated temperatures.^15,16^ In addition, LNMO undergoes complex phase transitions during (de) lithiation, resulting in volume changes and lattice strain that may lead to microcracking and particle pulverization over repeated cycles.^17,18^ The degree of Ni/Mn disorder adds complexity: higher disorder minimizes mechanical failure by promoting solid-solution behavior, but increases Jahn–Teller-active Mn^3+^, exacerbating interfacial instability *via* enhanced Mn dissolution.^19^

To mitigate these bulk structural issues, strategies like Cr-doping have been effectively employed to promote solid-solution phase transitions and reduce mechanical strain.^20,21^ Nonetheless, such bulk modifications are insufficient to resolve the interfacial instability at high voltages. Surface coating is a widely adopted strategy for interfacial stabilization.^22–25^ However, conventional coating strategies face a fundamental dilemma. On one hand, achieving an ideal continuous and uniform film is challenging with common synthesis routes due to thermodynamic and kinetic limitations inherent to solid–solid wetting.^23,26^ Even if successfully formed, a pinhole-free passivating layer—including those designed with Li-conductive phases—inevitably causes a kinetic penalty by introducing additional Li^+^ transport paths and barriers.^27^ Alternatively, the self-nucleation and unrestrained growth of a secondary phase often results in only large aggregates that are incapable of surface protection.

Herein, we overcome this coating dilemma by developing a thermodynamically driven self-assembly process on a Cr-doped LNMO platform. The introduction of trace zirconium to LiNi_0.49_Mn_1.49_Cr_0.02_O_4_ (LNMCO) crystallites triggers its spontaneous surface growth of tiny, uniform ZrO_2_ nano-islands. This phenomenon is rationalized as a Volmer–Weber growth mechanism, driven by the confluence of Zr immiscibility and significant lattice mismatch. This unique nano-island architecture provides robust surface stabilization and anchors a stable CEI layer without impeding Li^+^ transport, thus overcoming the limitations of conventional coatings. Through comprehensive theoretical and experimental analyses, we validate both the proposed formation mechanism and the outstanding performance of the resulting cathode under harsh operating conditions.

## 2 Results and discussion

### 2.1. Theoretical and experimental investigations for the formation of zirconia nano-islands

**Fig. 1:**
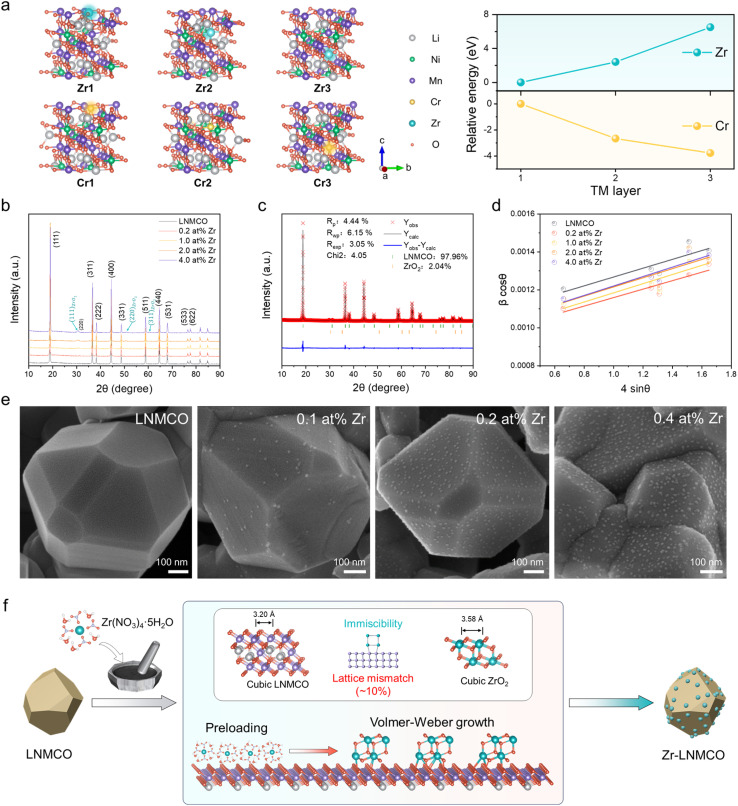
Theoretical and experimental analysis of Zr-modified LNMCO. (a) Structure models and calculated relative formation energies for Zr and Cr in the 1st, 2nd, and 3rd TM layers of the LNMO lattice. (b) XRD patterns of LNMCO and Zr-modified LNMCOs with increasing Zr content. (c) Rietveld refinement of the 4.0 at% Zr-LNMCO sample. (d) Williamson–Hall analysis showing consistent microstrain across all samples. (e) SEM micrographs revealing the formation of nano-islands on the surface of Zr-modified LNMCOs. (f) Schematic illustration for the mechanism of ZrO_2_ nano-island coating.

To theoretically understand the strategic design, we have performed density functional theory (DFT) calculations to elucidate the site preference and segregation behavior of Zr within the LNMO lattice in parallel with the typical dopant of Cr, based on formation energies across TM layers (Fig. 1a). The calculations reveal that Cr is energetically favored to incorporate into the bulk, whereas Zr possesses a high formation energy, driving its segregation to the surface. This theoretical prediction provides the fundamental rationale for our synthesis strategy. This predicted surface preference is further experimentally verified by subsequent structural and morphological analyses.

X-ray diffraction (XRD) patterns confirm that the underlying spinel structure is preserved upon Zr modification (Fig. 1b). Furthermore, Fourier-transform infrared (FTIR) and Raman spectra confirm that LNMCO is ordered one (P4_3_32),^7,28,29^ which remains intact after modification (SI Fig. S1 and S2). Notably, weak diffraction peaks corresponding to a cubic ZrO_2_ phase (*Fm*3̄*m*) emerge at Zr concentrations ≥1.0 at%. The formation of this high-temperature polymorph (typically above 2370 °C) at a modest 700 °C suggests a strong templating effect from the underlying cubic spinel substrate.^23^ Rietveld refinement of the XRD data quantifies the lattice parameters and the phase fractions for LNMCO modified by 4.0 at% Zr (Fig. 1c and Table S1). Despite the growth being templated to yield a structurally similar cubic phase (Fig. S3), a significant lattice mismatch (*f*) exists between the two oxides. Along their respective {111}, {110}, and {100} facets, the average *f* values are 10.61%, 9.98%, and 19.37% (Table S2). Williamson–Hall analysis (Fig. 1d) shows a negligible change in the microstrain of the bulk spinel phase after modification, further confirming that Zr segregates to the surface instead of being incorporated into the bulk lattice.

The combination of the bulk immiscibility and a large interfacial lattice mismatch creates a strong thermodynamic driving force for a Volmer–Weber (island) growth mechanism. Scanning electron microscopy (SEM) provides direct visual confirmation, revealing the formation of discrete nano-islands on the otherwise clean facets of the LNMCO crystallites upon the introduction of Zr (Fig. 1e). The surface density of these islands gradually increases with Zr concentration. Remarkably, even at a high dose of 2.0 at% Zr, the surface retains its nano-island architecture without coalescing into a continuous film or large aggregates (Fig. S4). As predicted, the truncated {100} facets, which possess the largest mismatch, show a much lower island density. Collectively, these results support a growth model (Fig. 1f) where thermodynamic forces determine the final morphology. A Zr-precursor, initially wetted onto the LNMCO surface, undergoes thermal decomposition and subsequent surface segregation due to its intrinsic immiscibility in the spinel lattice. There, a strong templating effect from the substrate directs its crystallization into a cubic ZrO_2_ phase under an unconventionally low temperature. Despite this shared symmetry, a large lattice mismatch then promotes the self-assembly of ZrO_2_ into the observed uniform nano-island architecture.

**Fig. 2:**
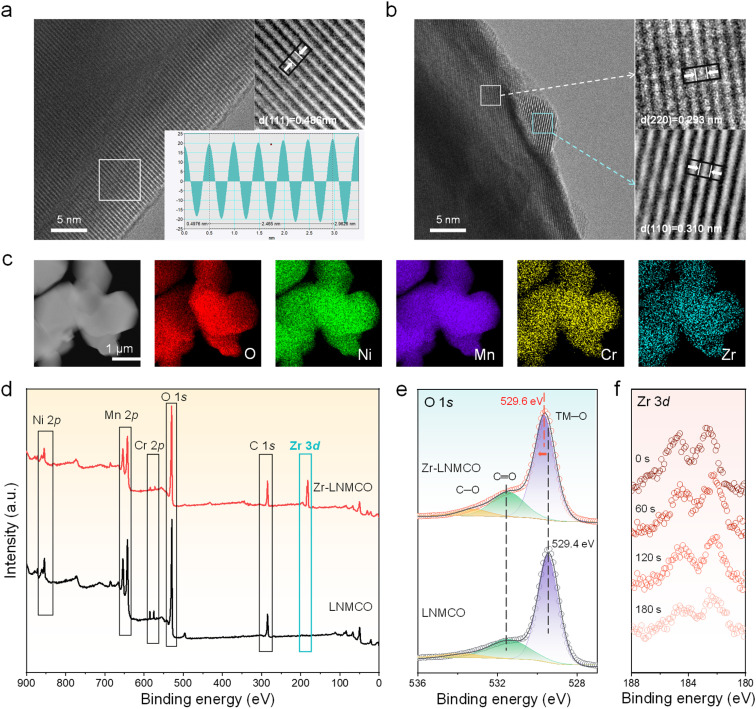
Nanoscale surface analysis and chemical characterization. (a and b) HRTEM images of LNMCO and Zr-LNMCO, with insets showing magnified views of the lattices. (c) EDS elemental mappings of the Zr-LNMCO. (d) XPS survey spectra comparing the elemental composition of LNMCO and Zr-LNMCO. (e) High-resolution O 1s spectra for the two cathode materials. (f) Zr 3d spectra of Zr-LNMCO as a function of etching time.

High-resolution transmission electron microscopy (HRTEM) has been employed to resolve the nano-island structure and composition. While the baseline LNMCO (Fig. 2a) exhibits a clean surface with continuous lattice fringes extending to the particle edge, the Zr-modified LNMCO (represented by 0.2 at% Zr coated LNMCO and denoted as Zr-LNMCO) reveals distinct nanoscale heterostructures (∼5 nm in size) decorating its surface (Fig. 2b). These nano-islands display a characteristic lattice spacing of 0.310 nm, attributable to the (110) planes of the cubic ZrO_2_ phase, which is clearly distinguishable from the underlying spinel lattice. This direct observation of crystalline ZrO_2_ nano-islands is also verified by additional imaging (Fig. S5). Elemental mapping *via* energy-dispersive X-ray spectroscopy (EDS) of Zr-LNMCO (Fig. 2c) shows a pronounced Zr signal outlining the particle, indicating a Zr-rich surface shell.

X-ray photoelectron spectroscopy (XPS) has also been used to analyze this surface enrichment and probe its effect on the local chemical environment. Survey spectra confirm the successful incorporation of Cr and the surface-specific modification by Zr; upon addition of Zr, the Cr 2p signal intensity is markedly reduced, while prominent Zr 3d peaks appear (Fig. 2d). Quantitative analysis reveals a surface atomic composition of Mn : Ni : Cr : Zr of 12.44 : 4.19 : 0.21 : 4.03. Notably, despite being added at only 10% of the Cr concentration, the Zr surface fraction is approximately 20-fold higher than that of Cr. This dramatic surface enrichment of Zr provides direct experimental validation for the DFT-predicted thermodynamic driving force for its segregation. High-resolution XPS spectra offer further insight into the surface chemistry (Fig. S6). The O 1s spectrum of Zr-LNMCO (Fig. 2e) exhibits a notable shift of the lattice oxygen (TM–O) peak to a higher binding energy (529.6 eV) compared to the baseline materials (529.4 eV). This shift reflects a decrease in electron density for the surface oxygen atoms, consistent with the formation of stronger, more covalent Zr–O bonds that enhance the oxidative stability of the cathode surface. Furthermore, depth-profiling XPS (Fig. 2f) has been applied to the Zr-LNMCO. The characteristic Zr 3d peaks gradually weakens during Ar^+^ etching, confirming the surface enrichment of Zr. Therefore, our combined theoretical and experimental results provide a cohesive picture of the nano-island formation. DFT calculations first predict the thermodynamic driving force for Zr surface segregation, a phenomenon visually confirmed by SEM, which reveals the spontaneous self-assembly of a uniform nano-island architecture. HRTEM analysis further resolves these islands as a crystalline, cubic ZrO_2_ phase and confirms the pristine nature of the underlying spinel lattice. Finally, surface-sensitive XPS and depth-profiling unequivocally verify the surface confinement of the Zr species, providing a robust, multi-technique validation of our proposed mechanism.

### 2.2. Electrochemical performance of nano-island-protected cathode materials

**Fig. 3:**
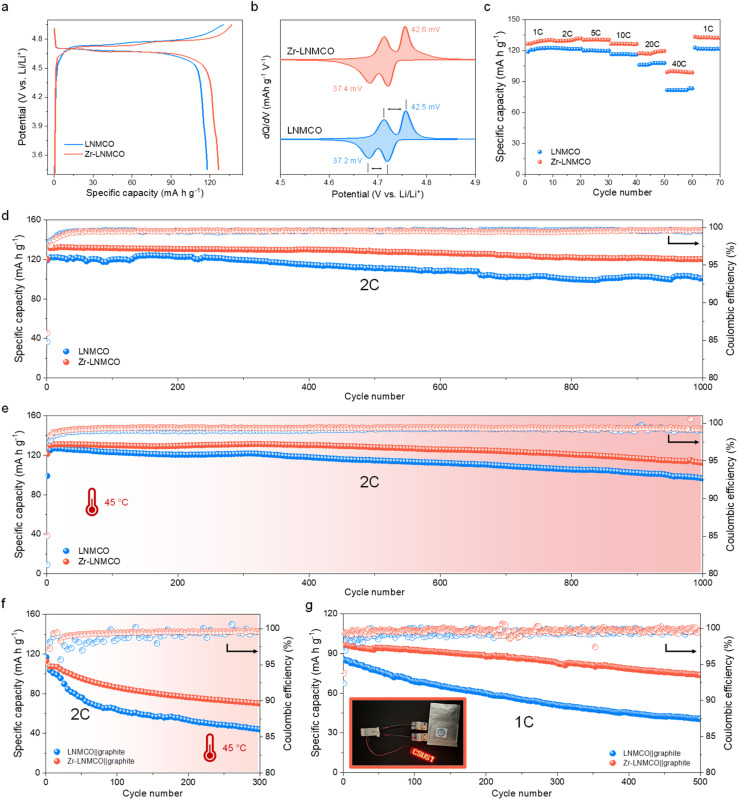
Electrochemical performance of LNMCO and Zr-LNMCO. (a) Initial charge–discharge curves of LNMCO and Zr-LNMCO at 1C. (b) d*Q*/d*V* profiles of the two cathodes. (c) Specific capabilities at different discharging rates. (d) Long cycling performance of the half cells at 2C. (e) Cycling performance of the cathodes at an elevated temperature of 45 °C. (f) Comparative cycling performance of LNMCO‖graphite and Zr-LNMCO‖graphite full cells at 2C at 45 °C. (g) Cycling performance of pouch cells at 1C.

The electrochemical behavior of the cathode materials has been first evaluated in the same half-cell configuration using Li metal foil as the counter electrode. Initial galvanostatic charge–discharge profiles at 1C (147 mA g^−1^) reveal that the Zr-LNMCO cathode delivers an improved specific capacity of 126.5 mA h g^−1^ with a high coulombic efficiency of 92.6%, surpassing the pristine LNMCO (117.8 mA h g^−1^, 90.4%) (Fig. 3a). The differential capacity (d*Q*/d*V*) curves (Fig. 3b) show that LNMCO and Zr-LNMCO electrodes display similar voltage separations between the Ni^2+^/Ni^3+^ and Ni^3+^/Ni^4+^ peaks (from 37.2 to 42.6 mV), which is in between the typical values for ordered and disordered spinels.^19,30^ The negligible Mn^3+^/Mn^4+^ redox signal near 4.0 V confirms the minimum Mn^3+^ content in the materials, in agreement with the unaltered bulk phase after modification.

The kinetic advantage endorsed by the nano-island architecture becomes evident in the rate capability (Fig. 3c). At an exceptionally high rate of 40C, the Zr-LNMCO electrode delivers an impressive 99.0 mA h g^−1^, substantially outperforming LNMCO (81.7 mA h g^−1^). Since the bulk Li^+^ diffusivity is comparable between LNMCO and Zr-LNMCO, this superior high-rate performance points directly to enhanced charge transfer kinetics at the nano-island-modified interface.^25,31^ To systematically deconvolve the effects of the bulk dopant and the surface modification, a comprehensive electrochemical comparison has been performed, with all results presented in Fig. S7. A direct comparison between pristine LNMO and the Cr-doped LNMCO baseline confirms the beneficial role of the dopant; LNMCO exhibits significantly improved rate capability, validating its use as an advanced platform for this study. Building upon this platform, a systematic study of Zr concentration then reveals that 0.2 at% is the optimal loading. At a lower concentration (0.1 at%), the sparser nano-island coverage provides insufficient surface modification, leading to suboptimal improvement. Conversely, a higher concentration (0.4 at%) results in a slight decrease in rate capability, which we attribute to increased interfacial impedance from an excessive loading of the ZrO_2_ phase. This demonstrates that the goal is not maximum coverage, but an optimized nano-island architecture that balances comprehensive surface protection with facile kinetics.

The significant impact of the surface modification is observed in long-term cycling stability. At a 2C rate, the Zr-LNMCO cathode demonstrates enhanced durability, retaining 90.8% of its capacity after 1000 cycles, in comparison to the LNMCO electrode (81.3%, Fig. 3d and S8). To place this high stability in context, a comprehensive comparison with recently reported Zr-modified LNMO cathodes^32–39^ is provided in Table S3. A side-by-side analysis reveals that the performance of our Zr-LNMCO cathode is among the best reported to date. This significant improvement is attributed to the unique nano-island architecture, which resolves the trade-off between surface passivation and interfacial kinetics. This stability is even more pronounced under thermally challenging conditions (45 °C), which are known to accelerate parasitic reactions at the high-voltage interface.^25,40^ Under this condition, the Zr-LNMCO cathode maintains an impressive 85.5% capacity after 1000 cycles, far exceeding the stability of LNMCO (76.1%) and highlighting the efficacy of the ZrO_2_ nano-islands in passivating the surface against thermal and electrochemical stress (Fig. 3e). To demonstrate practical viability, LNMCO and the optimized Zr-LNMCO cathodes have been paired with graphite anodes in a full-cell configuration. In coin cells cycled at 2C, the Zr-LNMCO‖graphite cell exhibits 78.2% capacity retention after 500 cycles, whereas the baseline LNMCO‖graphite cell retains 68.4% (Fig. S9). To assess performance under thermal stress, full cells were subjected to cycling at 45 °C after an initial 20 cycles at room temperature (Fig. 3f). The Zr-LNMCO‖graphite cell demonstrated excellent durability, retaining 67.1% after 280 heat-wave cycles, while the baseline cell rapidly degraded to 45.1% under the same conditions. This stability has been replicated in pouch cells, which retained 77.5% capacity after 500 cycles at 1C (*vs.* 50.4% for the baseline) (Fig. 3g). This level of cycle life in a full-cell configuration places our Zr-LNMCO among the top-performing LNMCO-based systems reported to date (Table S4).^24,40–48^

### 2.3. Mechanistic investigation of enhanced kinetics and stability

**Fig. 4:**
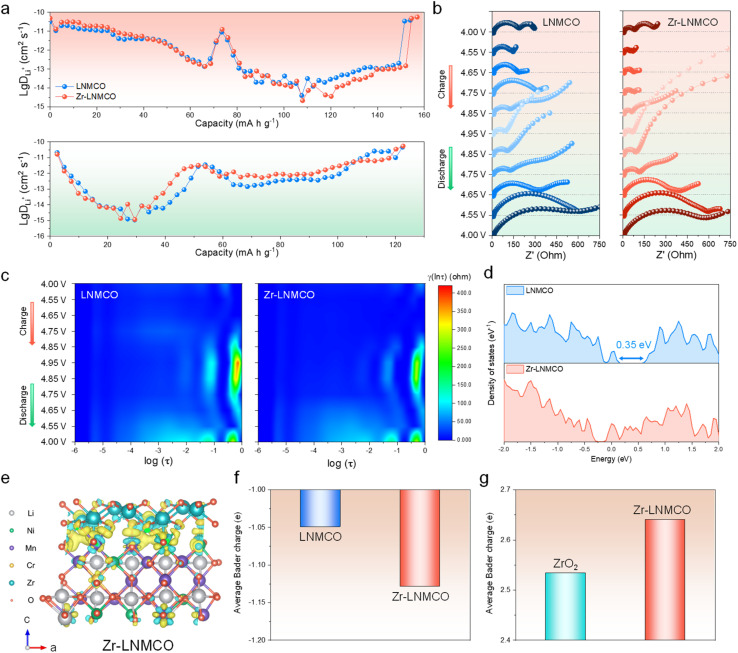
Mechanistic analyses of kinetic and stability enhancements. (a) Li^+^ diffusion coefficients of the cathodes during initial charge and discharge. (b) *In situ* EIS plots of the cathodes at the initial cycle. (c) Corresponding DRT results. (d) Total DOS near the Fermi level for LNMCO and Zr-LNMCO. (e) Differential charge density of the Zr-LNMCO heterostructure. (f) Average Bader charge of oxygen for LNMCO and Zr-LNMCO. (g) Average Bader charge of Zr in bulk and surface forms.

To understand the performance enhancement, Li^+^ diffusion dynamics of LNMCO and Zr-LNMCO electrodes have been investigated using the galvanostatic intermittent titration technique (GITT) method. Apparent Li-ion diffusion coefficients (*D*_Li_), reflecting both bulk transport and interfacial charge transfer kinetics,^49^ are determined from the time–potential profiles (Fig. S10) and are plotted in Fig. 4a for the initial charge–discharge cycle. As expected, two stages of *D*_Li_ variation can be observed, corresponding to two consecutive phase transitions (Li_1_ ↔ Li_0.5_ and Li_0.5_ ↔ Li_0_), where the second transition exhibiting slower kinetics.^50,51^ While both LNMCO and Zr-LNMCO exhibit similar *D*_Li_ at lower states of charge, a clear divergence is observed in the high-voltage region corresponding to the final, kinetically challenging delithiation stage. This indicates that the nano-island-modified surface facilitates more rapid ion transport during the Li_0.5_ → Li_0_ transition. Moreover, Zr-LNMCO shows clear advantage after 100 cycles, when the CEI is fully developed (Fig. S11), suggesting that the strategy effectively mitigates interfacial diffusion limitations.

*In situ* electrochemical impedance spectroscopy (EIS) has been employed to deconvolve the interfacial processes for LNMCO and Zr-LNMCO electrodes (Fig. 4b). To enhance accuracy beyond simplified equivalent circuit models, the distribution of relaxation times (DRT) method is employed to identify dominant electrochemical processes.^52^ Relaxation time statistics categorize high-frequency (−6 to −4 log scale) as solution resistance (*R*_s_), mid-frequency (−4 to −2) as cathode–electrolyte interfacial impedance (*R*_sf_), and low-frequency (−2 to 0) as charge transfer resistance (*R*_ct_). Initial impedances are comparable across samples (Fig. 4c). Upon charging, all the cathodes show dynamic *R*_ct_, due to evolving Li^+^ concentration gradients and CEI formation.^53^ Notably, Zr-LNMCO maintains a significantly lower and more stable *R*_ct_, especially at high states of charge (>4.7 V), where parasitic reactions are most severe. This enhanced charge-transfer kinetic at high potentials is likely the primary origin of the higher initial capacity observed for Zr-LNMCO. The nano-island architecture facilitates more complete and rapid lithium extraction during this final, kinetically-limited stage, thereby unlocking additional capacity that is otherwise inaccessible in the baseline material. This ability to maintain a low-impedance interface under high voltage is not only key to maximizing the initial capacity but is also fundamental to the excellent long-term cycling stability. After 100 cycles, the impedance of Zr-LNMCO is substantially lower than that of the counterpart (Fig. S12), affirming that the ZrO_2_ nano-island architecture facilitates fast kinetics and ensures long-term interfacial stability.

DFT calculations provide the fundamental rationale for these kinetic and stability improvements. Analysis of the electronic structure (Fig. 4d) reveals that Zr-LNMCO possesses higher density of states (DOS) near the Fermi level as well as absent energy gap (Fig. S13), indicating superior electronic conductivity for the surface heterostructure. This enhancement facilitates charge transfer between particles and could contribute to the observed reduction in *R*_ct_. Furthermore, to understand the enhanced electrochemical stability, we analyze the charge distribution at the ZrO_2_-LNMCO heterostructure. Differential charge density plots show significant charge accumulation at the interface, with electrons transferring from Zr to surface oxygen atoms (Fig. 4e). This is quantified by Bader charge analysis (Fig. 4f), which shows the average charge on surface oxygen atoms becomes more negative, changing from −1.05*e*^−^ in the baseline materials to −1.13*e*^−^ in Zr-LNMCO. Correspondingly, the average Bader charge of Zr in the heterostructure is higher than in its bulk ZrO_2_ form, confirming the charge transfer from Zr to O (Fig. 4g). This increased electron density signifies stronger Zr–O surface bonds, which effectively anchor the oxygen from cathode surface, thereby suppressing the parasitic reactions at high voltages.^54^

### 2.4. Post-cycling analysis of the stabilized interface

**Fig. 5:**
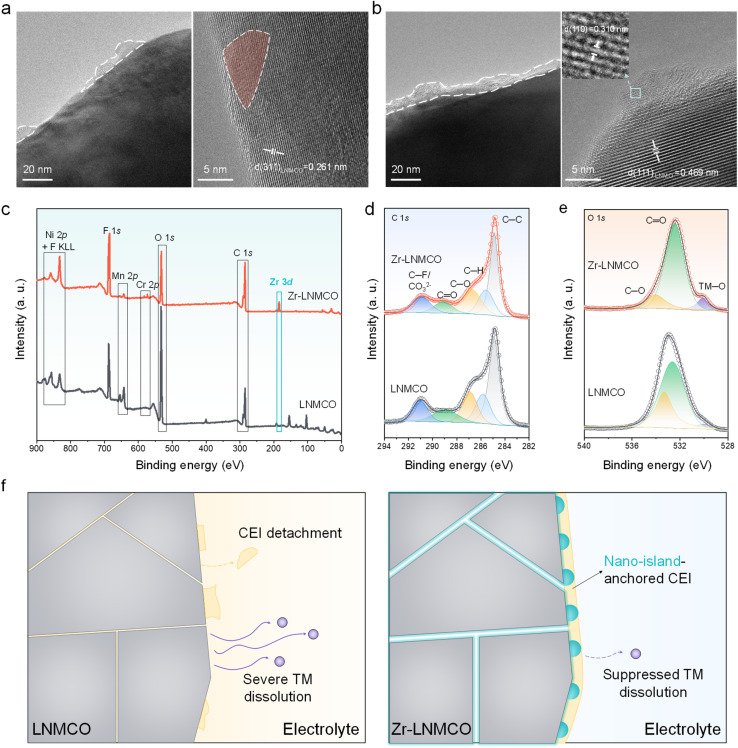
(a and b) HRTEM images of LNMCO and Zr-LNMCO after 1000 cycles. (c) XPS survey spectra of cycled LNMCO and Zr-LNMCO. (d and e) High-resolution C 1s and O 1s spectra of the cycled cathodes. (f) Schematic illustrations for the evolution of LNMCO and Zr-LNMCO over cycling.

To understand the mechanism behind the enhanced long-term stability, we have examined the structural and chemical evolution of the cathodes after 1000 cycles. HRTEM image of the cycled pristine LNMCO (Fig. 5a) reveals discontinuous amorphous deposits on the surface, indicative of a fragmentary and unstable CEI. This is likely a result of weak adhesion to the smooth crystal facets and/or degradation from HF attack.^55^ The underlying LNMCO lattice fringes appear blurred and discontinuous near the surface, suggesting structural damage from parasitic interfacial reactions. In contrast, the cycled Zr-LNMCO (Fig. 5b) exhibits a continuous CEI that fully encapsulates the cathode surface with the ZrO_2_ nano-islands embedded. Beneath this stable CEI, the LNMCO lattice fringes remain clear and well-defined, demonstrating preserved bulk crystallinity. The persistence of the ZrO_2_ nano-islands themselves, still tightly integrated with the LNMCO, is also confirmed. EDS analysis (Fig. S14) verifies Cr and Zr retention post-cycling.

Post-cycling XPS further elucidates the chemical evolution of the interface. After 1000 cycles, the Zr-LNMCO surface still shows a prominent Zr 3d signal, reaffirming the robust integration of the ZrO_2_ phase (Fig. 5c). Analysis of the C 1s spectra (Fig. 5d) reveals that the cycled Zr-LNMCO has a reduced intensity of carbon-containing decomposition products (C–O and C=O peaks) compared to the baseline LNMCO, indicating suppressed electrolyte degradation.^56^ This conclusion is reinforced by the O 1s spectra (Fig. 5e), where the Zr-LNMCO electrode shows a much weaker overall intensity and maintains of an evident lattice oxygen (TM–O) signal, signifying a thinner, less resistive CEI. The nano-island architecture also effectively suppresses TM dissolution, a key failure mode for Mn-contained spinel cathodes. The Mn 2p_3/2_ spectra reveal a lower total intensity and a smaller proportion of Mn^2+^, a species that is associated with MnF_2_ formation from HF attack, on the cycled Zr-LNMCO surface (Fig. S15).^57,58^ This finding is quantitatively verified by inductively coupled plasma mass spectrometry (ICP-MS) analysis of the lithium counter anodes after 1000 cycles (Fig. S16). The deposited ratios of Mn and Ni from the Zr-LNMCO cell account for only 30.3% and 30.1%, respectively, of those detected from the baseline LNMCO cell. Thus, the collective evidence reveals a synergistic stabilization mechanism, which is schematically presented in Fig. 5f. The process is initiated by the thermodynamically-driven surface segregation of trace Zr on LNMCO, leading to the self-assembly of a uniform ZrO_2_ nano-island architecture. These discrete nano-islands provide a multifunctional interface that resolves the fundamental dilemma of conventional coatings. They enhance surface electronic conductivity, contributing to improved interparicle conducivity. Meanwhile, the ZrO_2_ nano-islands stabilize surface lattice through interfacial Zr–O–TM bonding and create a textured surface that promotes the nucleation and adhesion of a robust, mechanically resilient CEI. Such unique surface architectures provide comprehensive passivation without creating a Li^+^ transport barrier, thereby ensuring the exceptional cycling stability even under harsh operating conditions.

## 3 Conclusion

In summary, this work establishes a highly effective strategy to resolve the critical interfacial instability haunting high-voltage LIBs by constructing a protective surface nano-island architecture on cathode surface. We have demonstrated that introducing trace zirconium to LNMCO crystallites triggers its spontaneous surface segregation and self-assembly into uniform ZrO_2_ nano-islands. The discrete nano-islands simultaneously enhance surface electronic conductivity, stabilize lattice oxygen, and anchor a robust cathode-electrolyte interphase without impeding Li^+^ transport. As a result, the nano-island-protected cathode exhibits much improved performance under demanding conditions, delivering a high-rate capacity of 99.0 mA h g^−1^ at 40C and retaining 85.5% of its capacity after 1000 cycles at 45 °C in half-cells. Critically, this stability translates to full-cell configurations, where a capacity retention of 67.1% is achieved after 280 cycles at 45 °C with a graphite anode, far surpassing the baseline material. This approach of creating a self-assembled, multifunctional nano-island interface provides an effective pathway for the development of next-generation, high-performance, and long-life batteries.

## Author contributions

G. Chu and Y. She conducted experiments, collected data, and wrote the original draft collaboratively. A. Huang, Q. Ye, Y. Deng, and Y. Sun contributed to the experimental design and data analysis. T. Lin, T. Schülli, L. Wang, and X. Zhu supervised the work and revised the manuscript. L. Wang and X. Zhu designed the project and funded it. All authors contributed to the manuscript. All authors read and approved the final manuscript.

## Conflicts of interest

There are no conflicts to declare.

## Supplementary Material

SC-OLF-D5SC07152F-s001

## Data Availability

The data that support the findings of this study is available from the corresponding authors upon reasonable request. Supplementary information: detailed methods and additional data/results. See DOI: https://doi.org/10.1039/d5sc07152f.
