# Supplementary material for: Volmer–Weber growth of nano-island heterostructures on spinel cathodes: a route to stable high-voltage lithium-ion batteries
Source: Chem Sci. 2025 Oct 31;16(48):23282–91. doi: 10.1039/d5sc07152f (PMC12590496; doi:10.1039/d5sc07152f)
Supplement: SC-016-D5SC07152F-s001 [file SC-016-D5SC07152F-s001.pdf]

Supplementary Information (SI) for Chemical Science.
This journal is © The Royal Society of Chemistry 2025

Supplementary Information

Volmer-Weber Growth of Nano-island Heterostructures on Spinel Cathodes: A Route to Stable High-Voltage Lithium-ion Batteries

Gui Chu#, Yuanqin She#, Aoyu Huang, Qingquan Ye, Yimei Deng, Tongen Lin*, Yongqi Sun, Tobias U. Schülli*, Lianzhou Wang *, and Xiaobo Zhu *

Experimental Section/Methods

Synthesis of LNMCO

The cathode materials were synthesized by an oxalate co-precipitation method [1]. First, 0.0206 mol of lithium acetate dihydrate (Aladdin, AR), 0.0298 mol of manganese acetate tetrahydrate (Aladdin, AR), 0.0098 mol of nickel acetate tetrahydrate (Aladdin, AR) and 0.0004 mol chromic acetate (Macalin, metal basis 99.9%) were dissolved in 100 mL of deionized water. Then, 0.0500 mol oxalic acid dihydrate (Rhawn, AR) was dissolved in 100 mL of ethanol. The metal acetate solution was then added dropwise to the oxalate solution under stirring. Then the suspension was heated and stirred at 90 °C until the evaporation of ethanol and water. The oxalate precursor was heated to 450 °C for three hours and grounded into a fine powder using a mortar. Afterwards, the powder was calcinated at 900 °C for 1 h and then 700 °C for 12 h to obtain the $LiNi_{0.49}Mn_{1.49}Cr_{0.02}O_4$ (LNMCO) products.

Surface Modification of LNMCO with ZrO_2 nano-islands

The ZrO_2-modified LNMCO (Zr-LNMCO) was prepared via a wet-milling process designed to ensure homogeneous precursor distribution. In a typical procedure, a precursor solution was first made by dissolving a stoichiometric amount of zirconium (IV) nitrate pentahydrate (Macklin, AR) (equivalent to 0.2 at% Zr relative to LNMCO, Macklin, AR) in anhydrous ethanol (4.0 mL). The as-prepared LNMCO powder (1.0 g) was then added to this solution. The resulting slurry was subjected to 20 minutes of continuous grinding in an agate mortar to ensure homogeneous mixing and complete drying. This powder was subsequently annealed in air at 700 °C for 1 h to form the final product. Samples with other Zr concentrations were prepared following the same procedure.

Material characterization

XRD patterns of the electrodes were collected from the X-ray diffractometer (D8 Advance, Bruker) under Cu K_α radiation (λ = 0.15406 nm). An infrared spectrometer (IRTracer-100, Shimadzu) was used to obtain FTIR spectra of the samples. Raman scattering was measured at room temperature with a Raman spectrometer (DXR-3xi, Thermo Fisher Scientific) with an excitation wavelength of 532 nm. The

morphologies and microstructures of the products were analyzed by an SEM (JSM-7900F, JEOL) and an EDS-equipped TEM (JEM-F200, JEOL). Surface compositions and chemical states of the electrodes were analyzed by an XPS (Thermo Scientific K-Alpha) with a monochromatic Al K_α excitation source. The calibrated binding energy for C 1*s* was 284.8 eV. To evaluate the protective effect of the electrode additive, cycled lithium electrodes were obtained from disassembled coin cells at charged states. The quantity of dissolved TM deposited on the lithium metal anode was quantified via ex-situ analysis. After 1000 cycles at 2C, half-cells were disassembled in a charged state inside an argon-filled glove box. The harvested lithium electrodes were then completely dissolved in aqua regia using a microwave digestion system. The resulting solutions were analyzed using an Agilent 7900 inductively coupled plasma mass spectrometer (ICP-MS) to determine the Mn and Ni content.

Electrochemical measurements

85 wt% active material, 10 wt% carbon black (acetylene black, Canrd), and 5 wt% polyvinylidene fluoride (PVDF, Solef) were mixed in the N-methyl-2-pyrrolidone (NMP, Aladdin) solution to form a slurry. Then, the paste was coated on an aluminum foil and vacuum-dried at 120 °C for 12 h. The dried film with an areal mass loading of 4.4 ± 0.6 mg cm^{-2} was cut into discs with a diameter of 1 cm. The electrochemical properties were tested by assembling the electrode discs into CR2032 button cells with lithium metal as the counter electrode in an argon-filled glove box (MIKROUNA). The electrolyte consisted of 1 mol/L $LiPF_6$ and 0.2 mol/L lithium difluoro(oxalato)borate (LiDFOB) dissolved in ethylene carbonate/diethyl carbonate/dimethyl carbonate (EC/DEC/DMC) (1:1:1, v/v/v). Celgard 2500 microporous membrane was used as the separator. Full cells were fabricated in both CR2032-type and pouch-type, where the negative electrodes were consisted of 85 wt% mesocarbon microbeads (graphite, Canrd), 10 wt% carbon black, and 5 wt% PVDF. The areal mass loading of the negative electrodes was 2.0 ± 0.3 mg cm^{-2}, corresponding to a negative to positive capacity ratio (N/P ratio) of 1.1 ± 0.05. In the fabrication of single-layer pouch cells, the positive electrodes were cut to a length of

46 mm and a width of 41 mm, while the negative electrodes were cut to a length of 48 mm and a width of 43 mm. 1 mL of electrolyte was used for each pouch cell. Charge-discharge tests were performed on a battery test system (CT-4008 T, NEWARE, China) under a room temperature of 26 ± 2 °C. For half-cells, the electrochemical window was 3.45–4.95 V (vs. Li/Li^+). For the full cells, the electrochemical window was 3.3–4.8 V. GITT measurements were conducted by charging and discharging at 0.1C for 10 min with 30 min rest intervals to allow the voltage to relax to steady state values. To evaluate the ion transport characteristics at the cathode-coating interface, we measured the D_{Li+} values of pristine LNMO and Zr-LNMCO samples using the commonly employed constant-current intermittent titration (GITT) method. The resulting time–voltage curves of the cathodes are displayed in Fig. S10. D_{Li} can be calculated from the following equation[2]:

$$D_{Li} = \frac{4}{\pi\tau}\left(\frac{m_B V_M}{M_B S}\right)^2\left(\frac{\Delta E_S}{\Delta E_\tau}\right)^2 \#(1)\#$$

where τ is the duration current pulse, m_B and M_B are the mass and molar mass of the cathode material, respectively, V_M is the molar volume, and S is the effective contact area of the cathode with the electrolyte. ΔE_S and ΔE_τ are the change in steady state potential and the total change in IR voltage drop subtracted during the current flow, respectively[3]. EIS spectra were acquired from an electrochemical workstation (Squidstat Plus, Admiral Instruments) in a frequency range of 1 MHz to 0.01 Hz.

Computational details

All our DFT calculations were performed using DS-PAW integrated in Device Studio program. [4] All our DFT calculations were performed using DS-PAW integrated in Device Studio program [2]. The Cr-doped LNMCO model and the Zr-LNMCO model were built on the cation ordered LNMO with $P4_332$ symmetry. Calculations were performed in the spin-polarized generalized gradient approximation (GGA) and in the GGA with Hubbard U correction (GGA+U). The Hubbard U values of 5.96, 5.0, 3.5, and 4.0 eV for Ni, Mn, Cr, and Zr were used to describe the strong correlation effect according to the previous studies [5, 6]. The Perdew-Burke-Ernzerhof exchange correlation [7] and a plane wave representation for the wave function with a cutoff

energy of 500 eV were used. The convergence criteria for energy and force were set to be 10^{-5} eV and 0.01 eV·Å^{-1}. The Monkhorst−Pack k point of 4 × 4 × 4 was adopted to relax the structures and 8 × 8 × 8 to calculate the density of states (DOS). Bader charge and differential charge density analyses were carried out to investigate charge transfer in the structures.

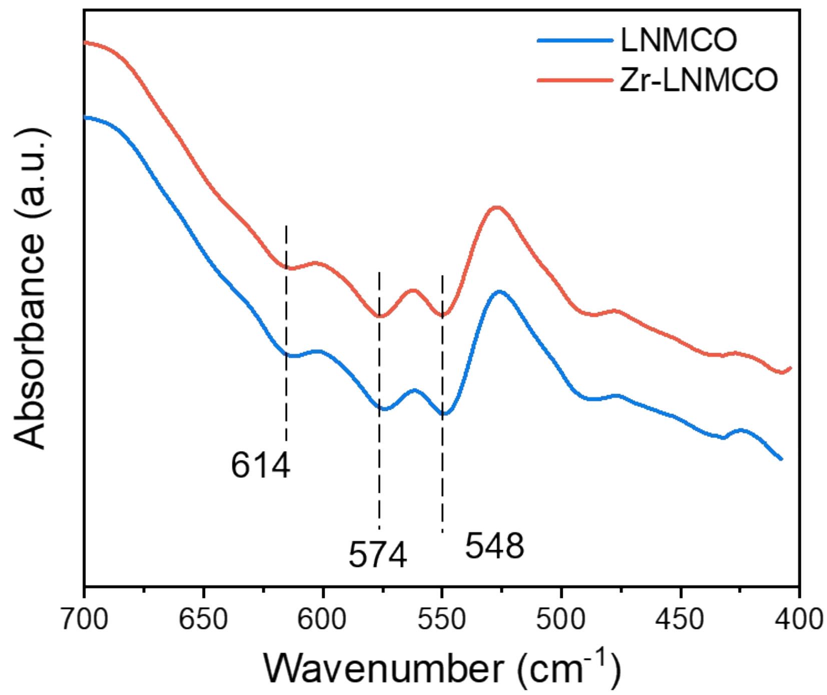

Figure S1. FTIR spectra of LNMCO and Zr-LNMCO

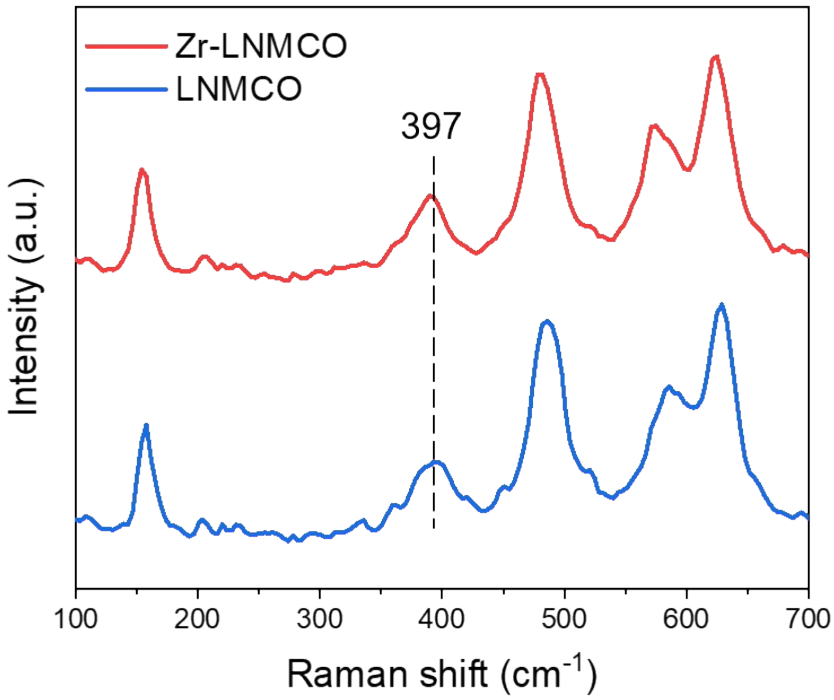

Figure S2. Raman spectra of LNMCO and Zr-LNMCO

Table S1 Rietveld refinement results of the XRD data for LNMCO modified by 4.0 at% Zr

Sample	Phase	*a* (Å)	*b* (Å)	*c* (Å)	*V* (Å^3)	Fraction	R_{wp}	Chi2
1	LNMCO	8.17387	8.17387	8.17387	546.114	97.96	6.15	4.05
	ZrO_2	5.09666	5.09666	5.09666	132.390	2.04		

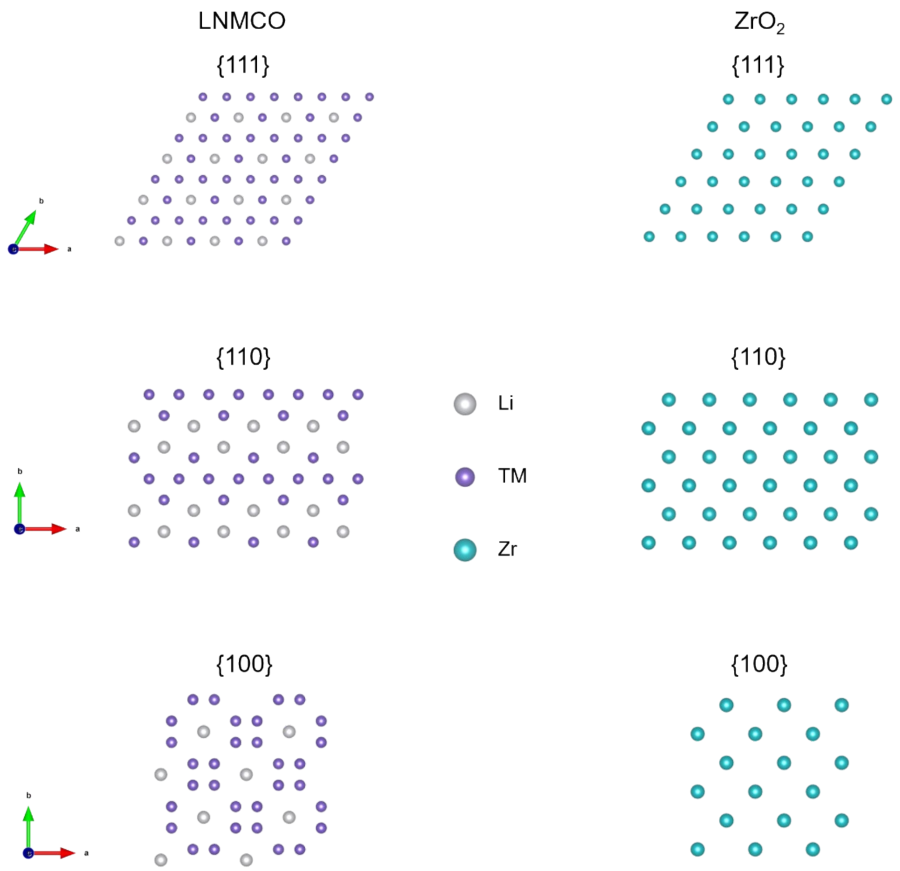

Figure S3. Epitaxial match between cubic LNMCO and cubic ZrO_2 from different planes (oxygen atoms are not shown).

Table S2. Calculated lattice mismach (*f*) between LNMCO and cubic ZrO_2 from typical crystal planes

LNMCO			ZrO_2			Minium *f*		
Crystal plane	Average interlayer spacing of oxygen in the *x*-direction (Å)	Average interlayer spacing of oxygen in the *y*-direction (Å)	Crystal plane	Interlayer spacing of oxygen in the *x*-direction (Å)	Average interlayer spacing of oxygen in the *y*-direction (Å)	*x*-direction (%)	*y*-direction (%)	Average (%)
{111}	3.20	3.20	{111}	3.58	3.58	10.61	10.61	10.61
{110}	2.89	3.87	{110}	2.53	3.58	12.46	7.49	9.98
{100}	2.04	2.04	{100}	2.53	2.53	19.37	19.37	19.37

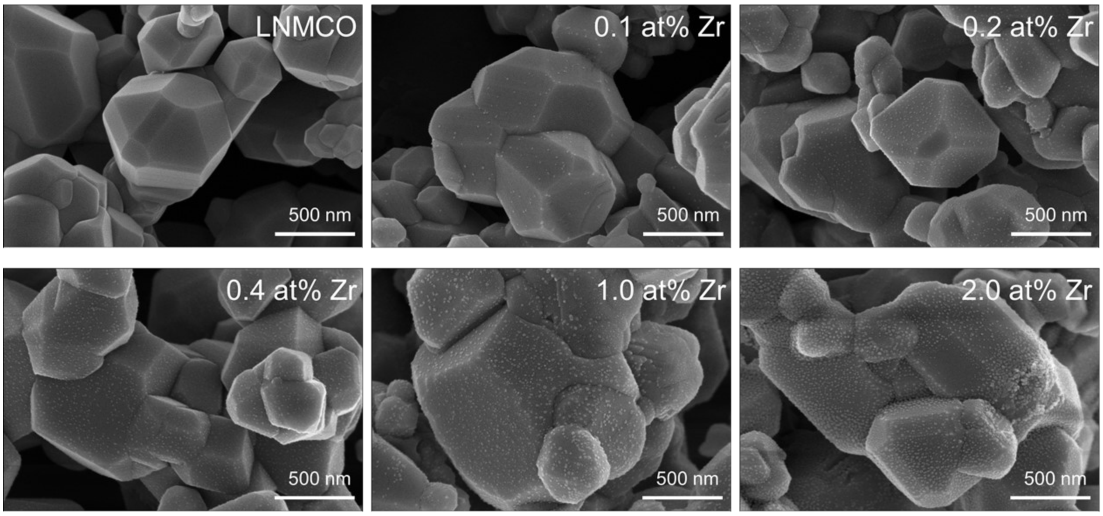

Figure S4. SEM images of LNMCO and Zr-modified LNMCO with different Zr contents.

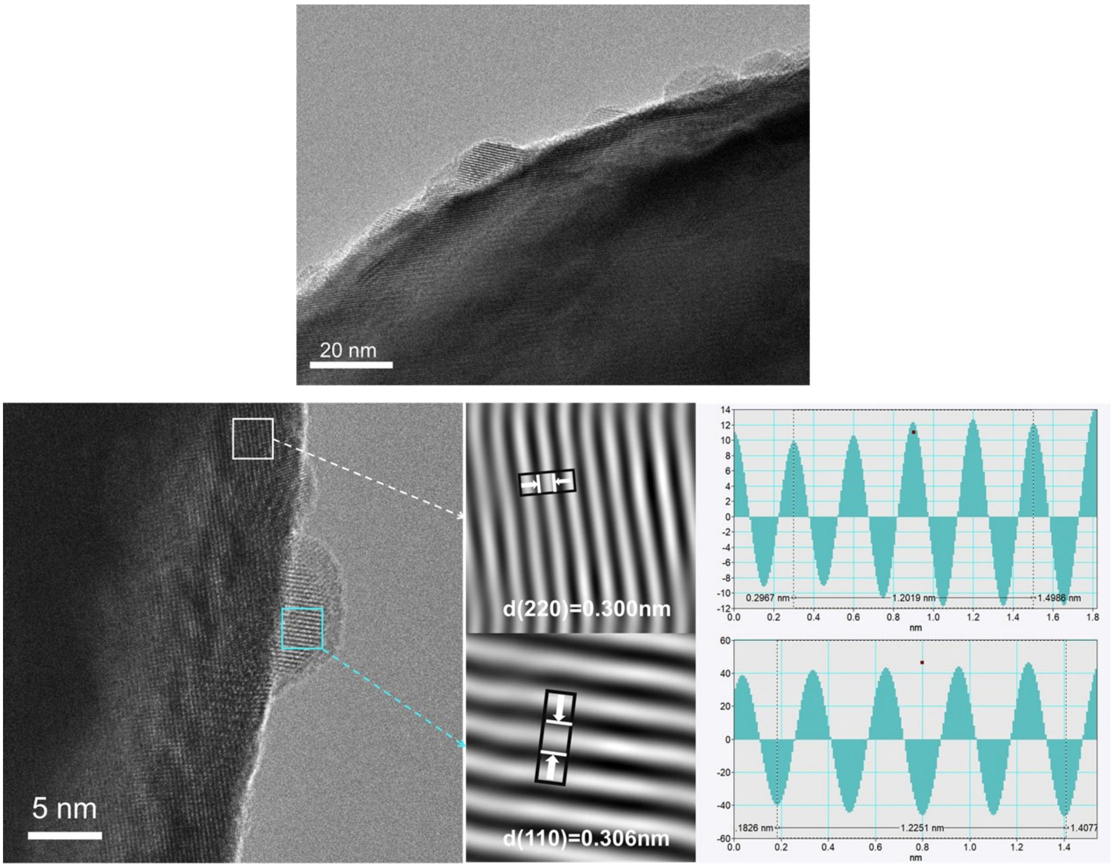

Figure S5. Additional HRTEM images of Zr-LNMCO.

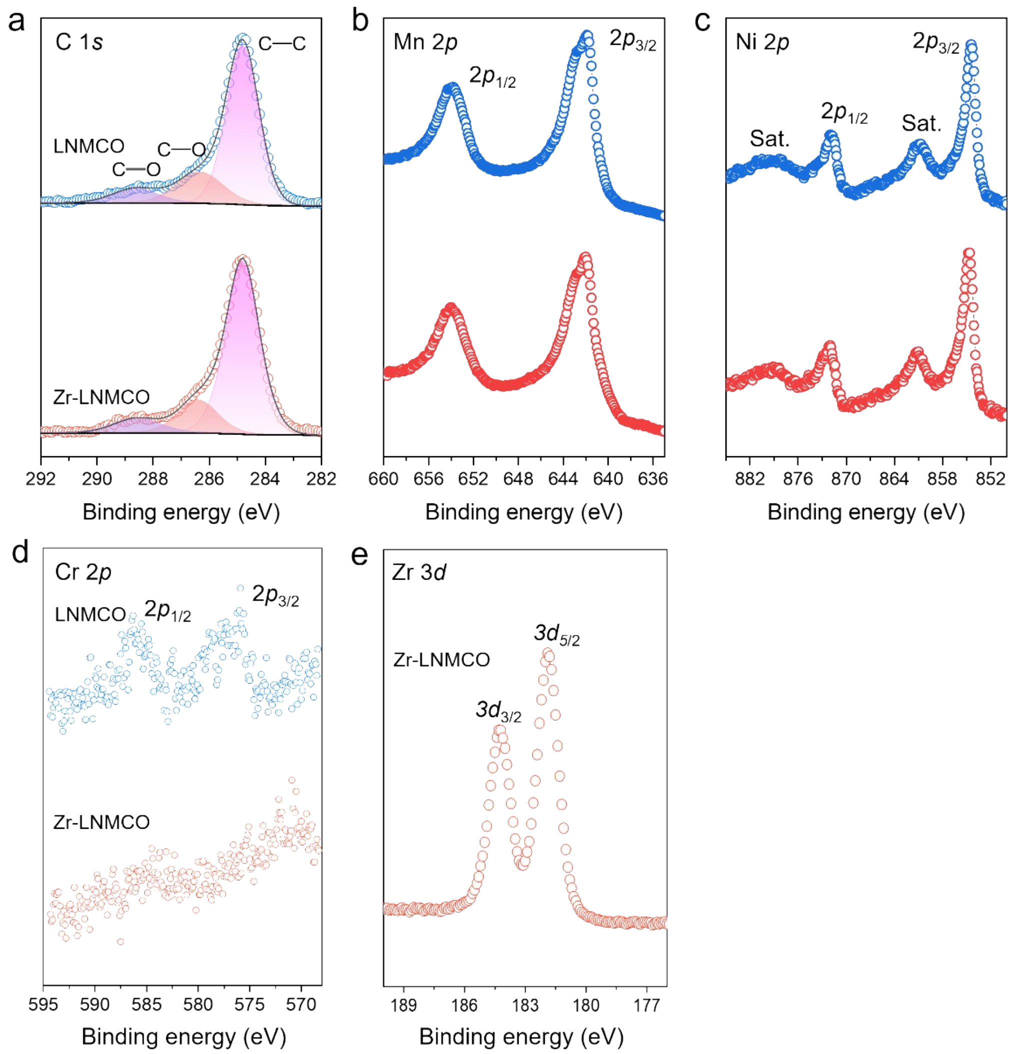

Figure S6. High-resolution C 1*s* (a), Mn 2*p* (b), Ni 2*p* (c), Cr 2*p* (d), and Zr 3*d* (e) spectra of LNMCO and Zr-LNMCO.

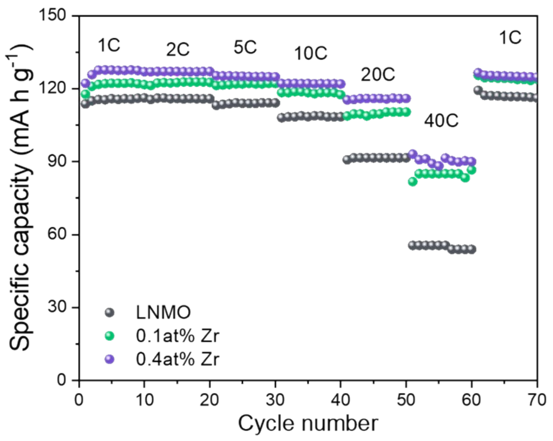

Figure S7. Rate performance of Zr-LNMCOs with different Zr contents.

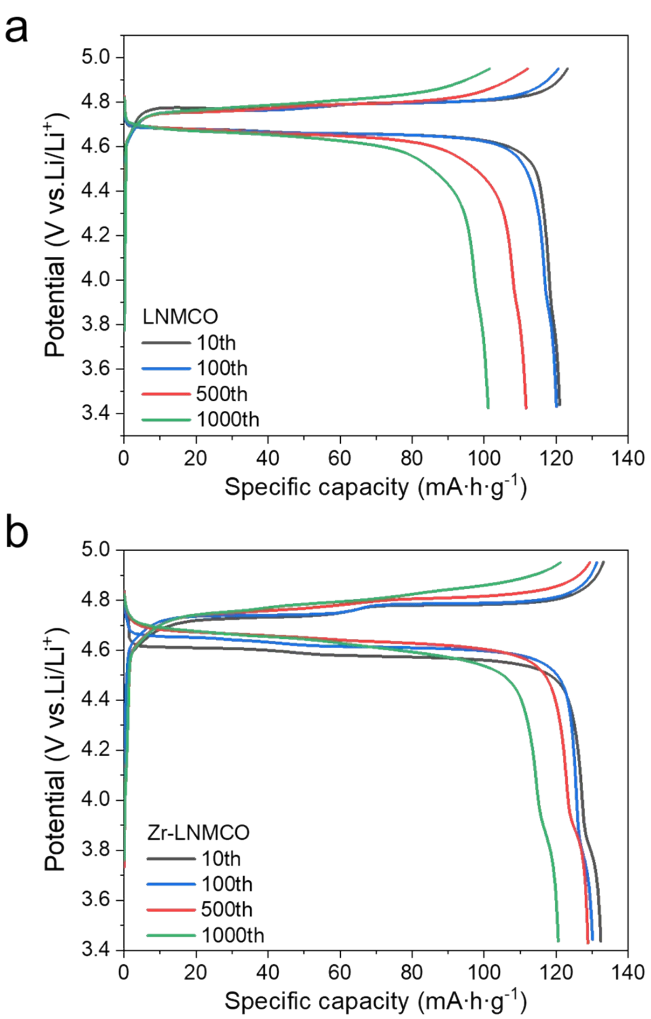

Figure S8. Charging/discharging curves of LNMCO (a) and Zr-LNMCO (b) at selected cycles.

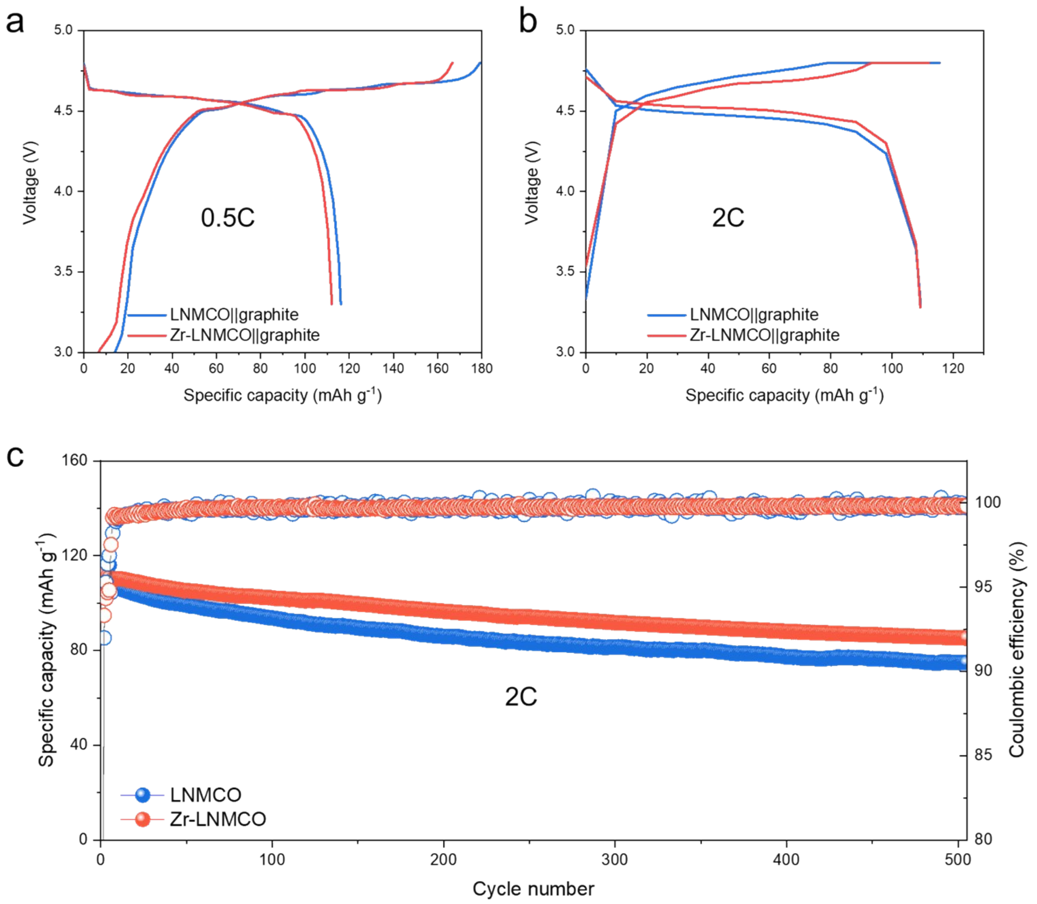

Figure S9. (a, b) Initial charge-discharge curves of LNMCO||graphite and Zr-LNMCO||graphite full cells at 0.5C (a) and 2C (b). (c) Cycling performance of the full cells.

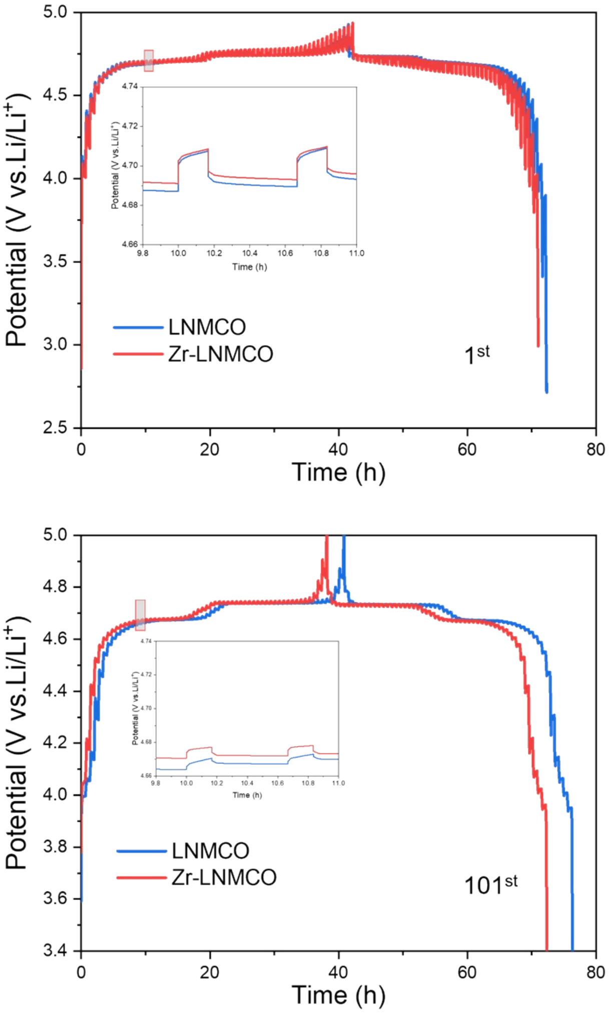

Figure S10. GITT profiles for LNMCO and Zr-LNMCO before and after 100 cycles.

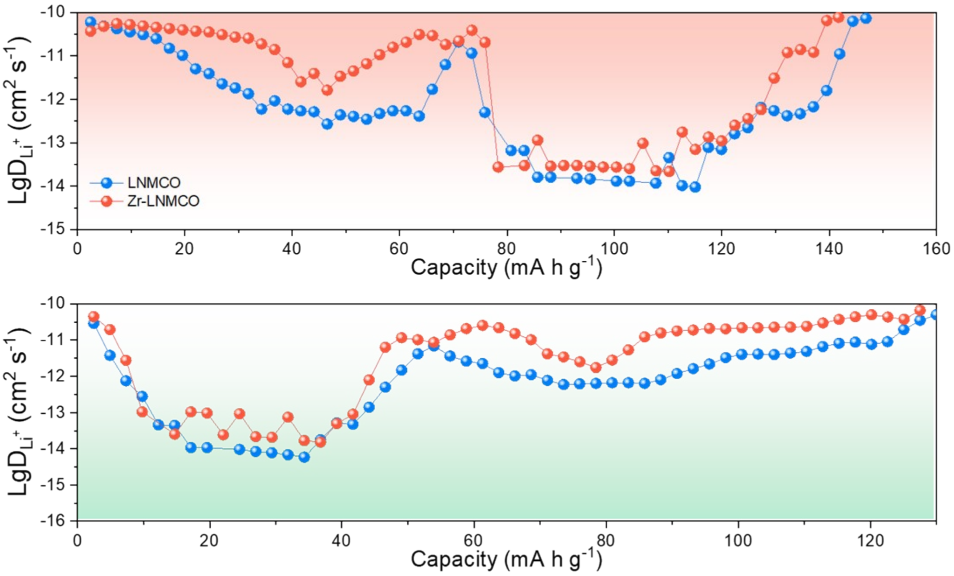

Figure S11. Comparison of Li^+ diffusion coefficients during charge-discharge of the cathodes after 100 cycles.

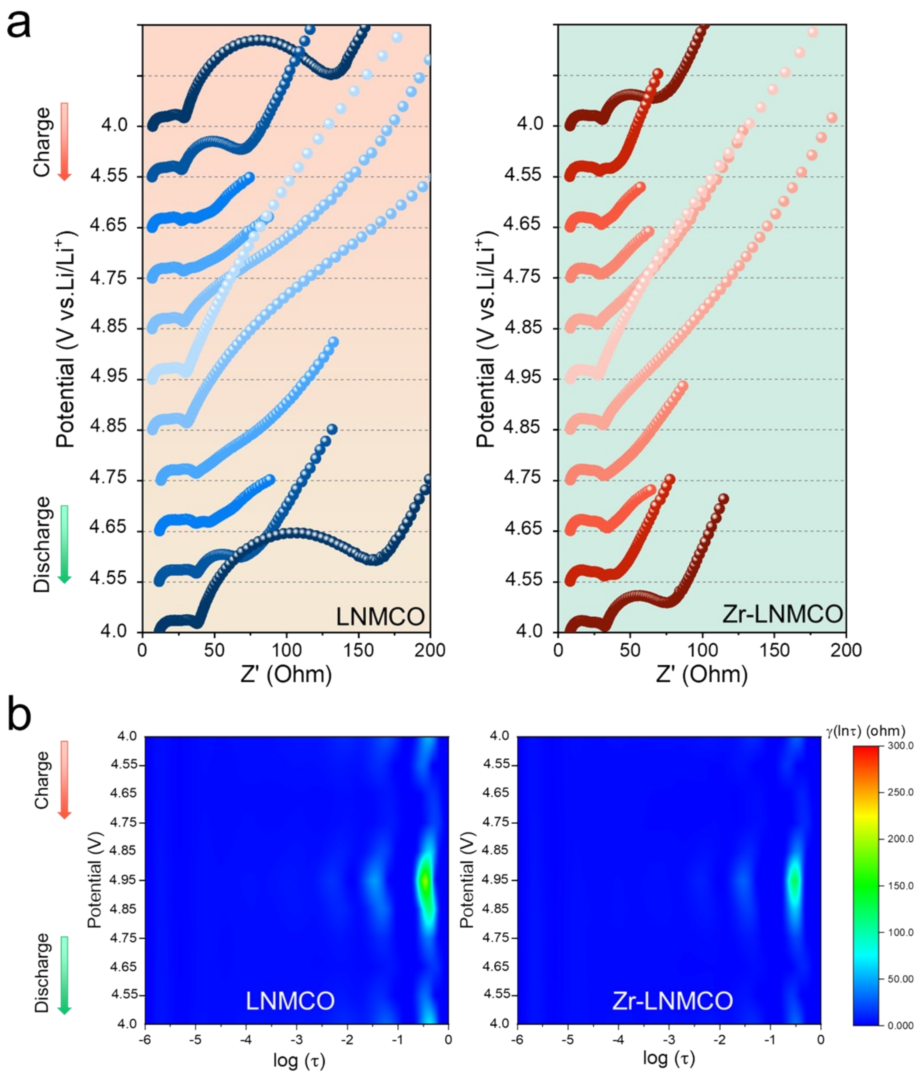

Figure S12. (a) *In-situ* EIS plots of the cathodes at the 101st cycle. (b) Corresponding DRT results.

Table S3. A comparison of our Zr-LNMCO with other recent works that utilized Zr as either a dopant or a coating layer for the modification of LNMO.

Key strategy	Electrochemical Performance
Zr and La co-doped LNMO[8]	84.5 mAh g^{-1} at 20C, 95.9% after 300 cycles at 20 C
Zr-doped $LiNi_{0.5}Mn_{1.49}Zr_{0.01}O_4$[9]	129.4 mAh g^{-1} at 1C, 95% after 200 cycles at 1 C
Li_2ZrO_3 coating and superficial Zr-doping[10]	130.2 mAh g^{-1} at 1C, 82.4% after 1000 cycles at 5 C
Zr-doped $Zr_{0.1}$-LNMO[11]	136.3 mAh g^{-1} at 0.1C, 76.8% after 100 cycles at 1 C
ZrO_2 -coated LNMO[12]	110 mAh g^{-1} (Charge rate 0.5C, discharge rate 40C), 85.6% after 1200 cycles (Charge rate 0.5C, discharge rate 40C)
ZrF_4-coated LNMO[13]	106 mAh g^{-1} at 2C, 95.5% after 120 cycles at 2C
Li_2ZrO_3-coated LNMO[14]	98.4 mAh g^{-1} at 10C, 77% after 1000 cycles at 1C
ZrO_2 and Li_3PO_4 composite-coated LNMO[15]	121.4 mAh g^{-1} at 1C, 88.5% after 100 cycles at 1C
This work	126.5 mAh g^{-1} at 1C, 90.8% after 1000 cycles at 2C

Table S4. A comparison of our Zr-LNMCO||graphite with some recently reported LNMO||graphite full cells at room temperature and elevated temperature.

Key strategy	Cycling performance
The use of a high-temperature-shock method to synthesis LNMO with surface oxygen vacancies[16]	97.3% after 50 cycles (1C, 25 °C)
Cu, Fe, and Cr co-doping of LNMO ($LiNi_{0.45}Cr_{0.0167}Fe_{0.0167}Cu_{0.0167}Mn_{1.5}O_4$)[17]	87.5% after 55 cycles (1C, 25 °C)
The use of pre-cycled graphite, pre-lithiated Fe-doped LNMO, and an electrolyte containing 30 wt.% fluoroethylene carbonate[18]	93% after 200 cycles (C/2-1C, 25 °C)
1,3,5-benzene-tricarboxylic acid coating of LNMO[19]	53% after 400 cycles (C/2-1C, 30 °C)
Zeolite coating of LNMO[20]	54% after 400 cycles (C/2-1C, 30 °C)
The use of potassium-nonafluoro-1-butanesulfonate as a slurry additive[21]	75.9% after 100 cycles (0.2C, 24 °C)
Zr-LNMCO||graphite (this work)	78.2% after 500 cycles (2C, 26 °C)
Construction of a LiF-rich artificial solid electrolyte interphase by pre-cycling the graphite in an electrolyte containing fluoroethylene carbonate[18]	50% after 100 cycles (C/2-1 C, 45°C)
1,3,5-benzene-tricarboxylic acid coating of LNMO[19]	43% after 200 cycles (C/2-1 C, 55°C)
Fluorinated carbonate electrolyte mitigating the interfacial reactions[22]	68 % after 100 cycles (C/10-C/3, 55°C)
Improve the LNMO/electrolyte interface through borate-based surface coating[23]	51% after 200 cycles (C/2-1 C, 45°C)
Tris(trimethylsilyl) phosphite and lithium difluoro(oxalato)borate as electrolyte additives[24]	60% after 140 cycles (0.3 C, 55°C)
5-Trifluoromethylpyridine-trimethyl lithium borate as electrolyte additives[25]	64% after 45 cycles (C/5-C/2, 55°C)
Zr-LNMCO||graphite (this work)	73.0% after 180 cycles (2C, 45 °C)

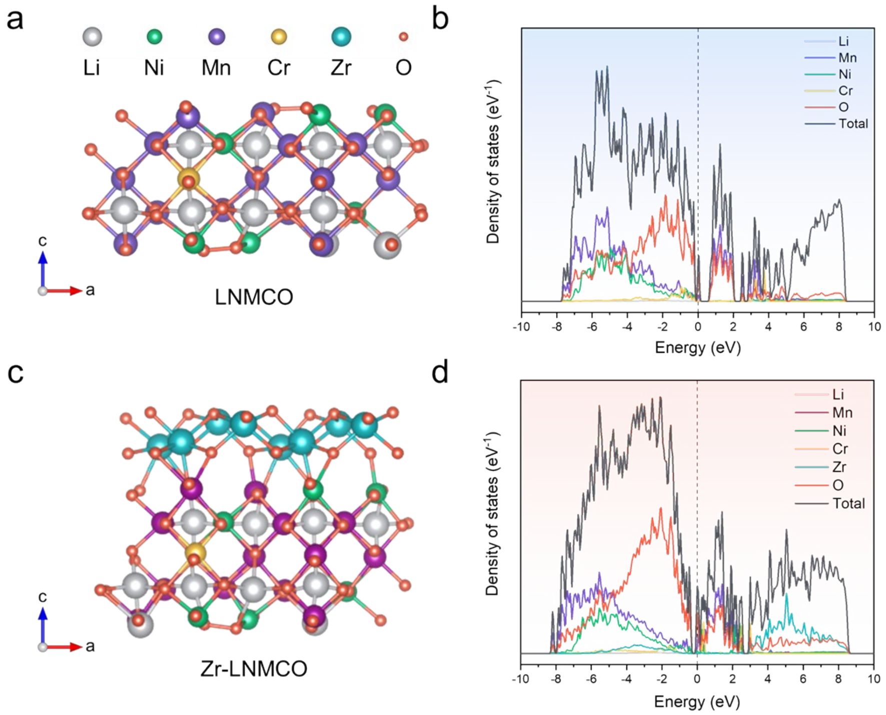

Figure S13. Relaxed structural models and projected DOS of LNMCO (a, b), Zr-LNMCO (c, d), the black dashed line shows the Fermi energy level.

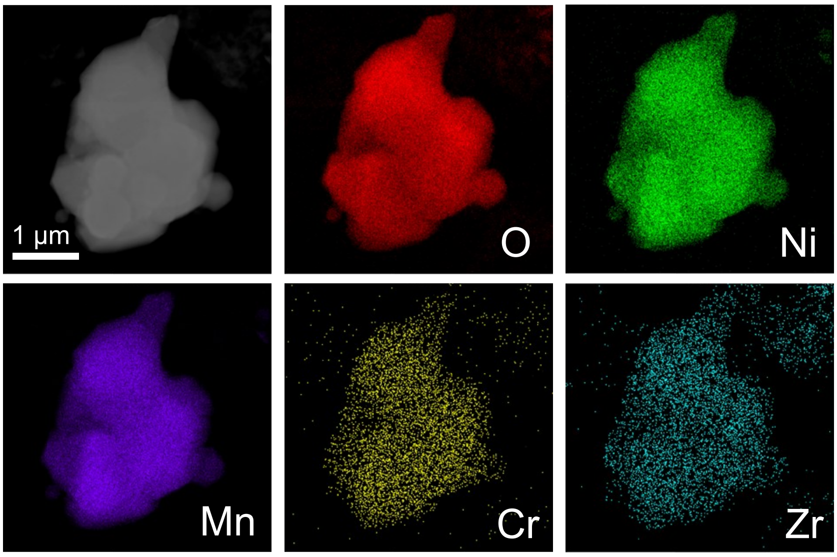

Figure S14. EDS elemental mappings of the Zr-LNMCO sample after 1000 cycles.

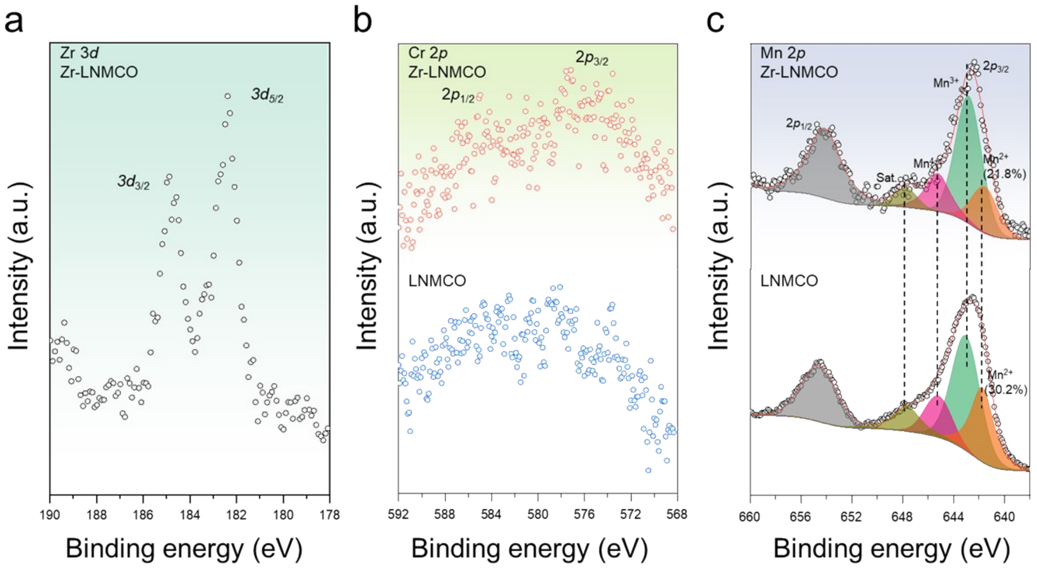

Figure S15. High-resolution Zr 3*d* (a), Cr 2*p* (b), and Mn 2*p* (c) spectra of cycled LNMCO and/or Zr-LNMCO.

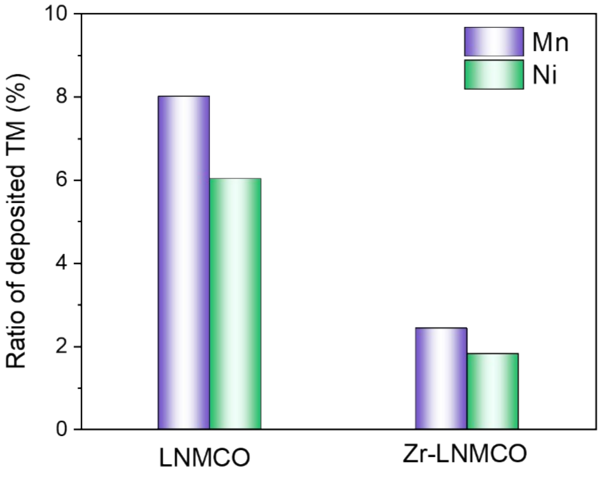

Figure S16. Ratios of deposited manganese and nickel on the counter Li electrodes relative to the manganese and nickel in the cathode material after 1000 cycles at 2C.

Supplementary References

1. Z. Tong, Q. Ye, Y. Deng, Q. She, A. Huang, J. Xu and X. Zhu, *Journal of Alloys and Compounds*, 2023, **937**, 168544.
2. M. Xu, M. Yang, M. Chen, L. Gu, L. Luo, S. Chen, J. Chen, B. Liu and X. Han, *Journal of Energy Chemistry*, 2023, **76**, 266-276.
3. M. Kuenzel, G.-T. Kim, M. Zarrabeitia, S. D. Lin, A. R. Schuer, D. Geiger, U. Kaiser, D. Bresser and S. Passerini, *Materials Today*, 2020, **39**, 127-136.
4. B. P. E, *Physical review. B, Condensed matter*, 1994, **50**, 17953-17979.
5. M.-C. Yang, B. Xu, J.-H. Cheng, C.-J. Pan, B.-J. Hwang and Y. S. Meng, *Chem. Mat.*, 2011, **23**, 2832-2841.
6. Y. Gao, X. Wang, J. Ma, Z. Wang and L. Chen, *Chem. Mat.*, 2015, **27**, 3456-3461.
7. J. P. Perdew, K. Burke and M. Ernzerhof, *Physical Review Letters*, 1996, **77**, 3865-3868.
8. W. Liu, Q. Shi, Q. Qu, T. Gao, G. Zhu, J. Shao and H. Zheng, *Journal of Materials Chemistry A*, 2017, **5**, 145-154.
9. S. Feng, X. Kong, H. Sun, B. Wang, T. Luo and G. Liu, *Journal of Alloys and Compounds*, 2018, **749**, 1009-1018.
10. J. Zhang, G. Sun, Y. Han, F. Yu, X. Qin, G. Shao and Z. Wang, *Electrochimica Acta*, 2020, **343**, 136105.
11. Y.-W. Song, J. Lee, Y. Jung, M.-Y. Kim and J. Lim, *Langmuir*, 2024, **40**, 22803-22811.
12. U. Nisar, R. Amin, R. Essehli, R. A. Shakoor, R. Kahraman, D. K. Kim, M. A. Khaleel and I. Belharouak, *Journal of Power Sources*, 2018, **396**, 774-781.
13. Y. Li, D. Wang, T. Xu, M. Wu, D. Pan, H. Zhao and Y. Bai, *Solid State Ionics*, 2018, **324**, 7-12.
14. R. Zhao, L. Li, Y. P. Li, T. H. Xu, D. Pan, C. Y. Yu, H. L. Zhao and Y. Bai, *Applied Physics Letters*, 2020, **116**, 021601.

15. G. Kaur, F. Nesvaderani, L. Hadidi, D. Dunn, S. Campbell and B. D. Gates, *ACS Applied Energy Materials*, 2022, **5**, 14335-14352.
16. H. Jiang, C. Zeng, W. Zhu, J. Luo, Z. Liu, J. Zhang, R. Liu, Y. Xu, Y. Chen and W. Hu, *Nano Research*, 2024, **17**, 2671-2677.
17. T. Tian, L.-L. Lu, Y.-C. Yin, Y.-H. Tan, T.-W. Zhang, F. Li and H.-B. Yao, *Small*, 2022, **18**, 2106898.
18. Z. Cui, F. Zou, H. Celio and A. Manthiram, *Advanced Functional Materials*, 2022, **32**, 2203779.
19. S. Maiti, H. Sclar, J. Grinblat, M. Talianker, Y. Elias, X. Wu, A. Kondrakov and D. Aurbach, *Small Methods*, 2022, **6**, 2200674.
20. S. Maiti, H. Sclar, X. Wu, J. Grinblat, M. Talianker, A. Kondrakov, B. Markovsky and D. Aurbach, *Energy Storage Materials*, 2023, **56**, 25-39.
21. J. Zhang, L. Cao, J. Li, M. Yang, J. Yu, Y.-J. Cheng, Y. Huang and Y. Xia, *Energy Storage Materials*, 2024, **64**, 103060.
22. W. Yao, Y. Li, M. Olguin, S. Bai, M. A. Schroeder, W. Li, A. Liu, N. R. Park, B. Bhamwala, B. Sayahpour, G. Raghavendran, O. Borodin, M. Zhang and Y. S. Meng, *Next Energy*, 2024, **4**, 100136.
23. U. Nisar, J. Bansmann, M. Hebel, B. Reichel, M. Mancini, M. Wohlfahrt-Mehrens, M. Hölzle and P. Axmann, *Chemical Engineering Journal*, 2024, **493**, 152416.
24. A. Jamal, G. D. Salian, A. Mathew, W. Wahyudi, R. P. Carvalho, R. Gond, S. K. Heiskanen, D. Brandell and R. Younesi, *ChemElectroChem*, 2023, **10**, e202300139.
25. Z. Xia, K. Zhou, X. Lin, Z. Xie, Q. Chen, X. Li, J. Cai, S. Li, H. Wang, M. Xu and W. Li, *Journal of Energy Chemistry*, 2024, **91**, 266-275.
